# A lightweight fetal distress-assisted diagnosis model based on a cross-channel interactive attention mechanism

**DOI:** 10.3389/fphys.2023.1090937

**Published:** 2023-03-06

**Authors:** Yanjun Deng, Yefei Zhang, Zhixin Zhou, Xianfei Zhang, Pengfei Jiao, Zhidong Zhao

**Affiliations:** ^1^ School of Electronics and Information, Hangzhou Dianzi University, Hangzhou, China; ^2^ School of Cyberspace Security, Hangzhou Dianzi University, Hangzhou, China

**Keywords:** fetal distress, fetal heart rate, lightweight model, attention mechanism, wavelet packet coefficient

## Abstract

Fetal distress is a symptom of fetal intrauterine hypoxia, which is seriously harmful to both the fetus and the pregnant woman. The current primary clinical tool for the assessment of fetal distress is Cardiotocography (CTG). Due to subjective variability, physicians often interpret CTG results inconsistently, hence the need to develop an auxiliary diagnostic system for fetal distress. Although the deep learning-based fetal distress-assisted diagnosis model has a high classification accuracy, the model not only has a large number of parameters but also requires a large number of computational resources, which is difficult to deploy to practical end-use scenarios. Therefore, this paper proposes a lightweight fetal distress-assisted diagnosis network, LW-FHRNet, based on a cross-channel interactive attention mechanism. The wavelet packet decomposition technique is used to convert the one-dimensional fetal heart rate (FHR) signal into a two-dimensional wavelet packet coefficient matrix map as the network input layer to fully obtain the feature information of the FHR signal. With ShuffleNet-v2 as the core, a local cross-channel interactive attention mechanism is introduced to enhance the model’s ability to extract features and achieve effective fusion of multichannel features without dimensionality reduction. In this paper, the publicly available database CTU-UHB is used for the network performance evaluation. LW-FHRNet achieves 95.24% accuracy, which meets or exceeds the classification results of deep learning-based models. Additionally, the number of model parameters is reduced many times compared with the deep learning model, and the size of the model parameters is only 0.33 M. The results show that the lightweight model proposed in this paper can effectively aid in fetal distress diagnosis.

## 1 Introduction

Fetal distress is a syndrome of respiratory and circulatory insufficiency caused by intrauterine fetal hypoxia during labor and is closely associated with changes in fetal heart rate signals (Blickstein and Green, 2007; Spairani et al., 2022). Fetal distress may cause hypoxic-ischemic encephalopathy and eventually leading to cerebral palsy or perinatal death (Bobrow and Soothill, 1999). Early detection and diagnosis of fetal distress can help prevent damage to the vital organs of the fetus prior to delivery. Therefore, it is important to enhance intrauterine fetal status monitoring during pregnancy to ensure the safety of the fetus and the pregnant woman. The most common method for monitoring fetal status in clinical practice is CTG monitoring (Grivell et al., 2015). The CTG signal consists of the FHR curve and uterine contraction (UC) curve. Through CTG monitoring, doctors can detect fetal distress in time so that they can take effective treatment measures to protect the health of the fetus. However, the diagnosis is too dependent on physician experience and interobserver disagreement when interpreted by the physician’s naked eye alone (Bernardes et al., 1997; Palomaki et al., 2006). Therefore, there is an increased incidence of unnecessary cesarean section due to subjective physician error (Abdulhay et al., 2014; Marques et al., 2019).

With the development of artificial intelligence technology, scholars worldwide are committed to developing fetal health-assisted diagnosis systems based on machine learning and deep learning to help healthcare professionals analyze CTG signals objectively and correctly. Barquero-Perez et al. (2017); Spilka et al. (2014); Georgoulas et al. (2017); Yilmaz. (2016) used normalized compression distance, random forest (RF), support vector machine (SVM), and artificial neural network (ANN) classification algorithms, respectively, to classify CTG signals for fetal distress problems and achieved good results. Zhao et al. (2018) extracted 47 features from different domains (morphological, time domain, frequency domain and non-linear domain) and selected Decision Tree, SVM and adaptive boosting, respectively, for fetal acidosis classification. Comert et al. (2018) used short-time Fourier transform (STFT) to obtain 2-D images and combined it with transfer learning and convolutional neural networks to predict fetal distress (Liu et al., 2021). proposed an attention-based CNN-BiLSTM hybrid neural network enhanced with features of discrete wavelet transformation, obtaining an average sensitivity, specificity and quality index of 75.23%, 70.82%, and 72.93%, respectively. Zhao et al. (2019) used recurrence plot to convert one-dimensional FHR to two-dimensional and fed into convolutional neural network to obtain 98.69% accuracy in fetal distress classification. Baghel et al. (2022) obtained 99.09% classification accuracy by performing direct 1-D convolutional operations on the FHR signal after Butterworth filtering. Although the abovementioned classification models based on machine learning and deep learning achieve better results, the complexity of the model and the large number of parameters take up large computational resources, which leads to the model being highly dependent on the performance of the device hardware and difficult to deploy to the terminal for generalized application.

Lightweight models and miniaturization have become a trend in many application scenarios, so an increasing number of academics are focusing on lightweight network models that can be deployed and run directly on mobile devices. The MobileNet series (Howard et al., 2017; Sandler et al., 2018; Howard et al., 2019) and ShuffleNet series (Ma et al., 2018; Zhang et al., 2018) of lightweight networks currently have good performance in the target detection and image classification field. MobileNet model is a lightweight deep neural network proposed by Google for embedded devices, using the core idea of depthwise separable convolution. ShuffleNet model is a neural network structure designed for devices with limited computational resources, mainly using pointwise group convolution and channel shuffle. Lightweight models are also beginning to make their mark in the medical signaling field. Cao et al. (2021) proposed a multichannel lightweight model with each channel integrating multiple heterogeneous convolutional layers to obtain multilevel features for classifying myocardial infarction with an accuracy rate of 96.65%. Zheng et al. (2021) trained MobileNetV1 and MobileNetV2 models by migration learning for pterygium diagnosis in the eye and compared them with the classical model and found that MobileNetV2 obtained better results with a model size of only 13.5 M. Chen et al. (2022) used the lightweight networks MobileNetV1, MobileNetV2, and Xception to classify cervical cancer cells and used knowledge distillation for accuracy improvement. Among them, Xception matched the accuracy of the large network Inception-ResNetV2, while the model size was only 40%. The lightweight network model effectively reduces the number of model parameters and opens up a method for promoting a low-cost operating model. However, the feature extraction ability and the network classification accuracy still need to be further improved.

Aiming at the complexity and considerable computation in existing deep learning-based fetal distress algorithm models, this paper introduces a lightweight network architecture to design a lightweight fetal distress-assisted diagnosis network based on FHR. Additionally, to further improve the feature extraction ability and classification effect of the network, the attention mechanism is incorporated into the lightweight network to build a lightweight network unit (ECA-Shuffle) based on the cross-channel interactive attention mechanism. The main contributions of this paper are as follows.

(1) The matrix feature map based on wavelet packet coefficients is constructed to refine the FHR signal in multiple frequency bands and used as input to the model. Different wavelet basis functions are selected to generate multiple feature maps to vote on the sample classification results.
(2) The cross-channel interactive attention module is embedded in the tail of the ShuffleNet-V2 base unit to generate an ECA-Shuffle unit to achieve effective multichannel feature fusion without dimensionality reduction.
(3) A lightweight fetal distress-assisted diagnosis network based on the FHR signal, LW-FHRNet, is proposed. Conventional convolution with ECA-Shuffle units ensures effective channel feature fusion while reducing model complexity and enhances the model’s ability to classify fetal distress.

The rest of the paper is presented below. Section 2 describes the overall scheme in detail. Section 3 describes the database, experimental setup and results in detail. Section 4 discusses and analyzes the performance of the proposed model. The final section contains conclusions and future work.

## 2 Materials and methods

**FIGURE 1:**
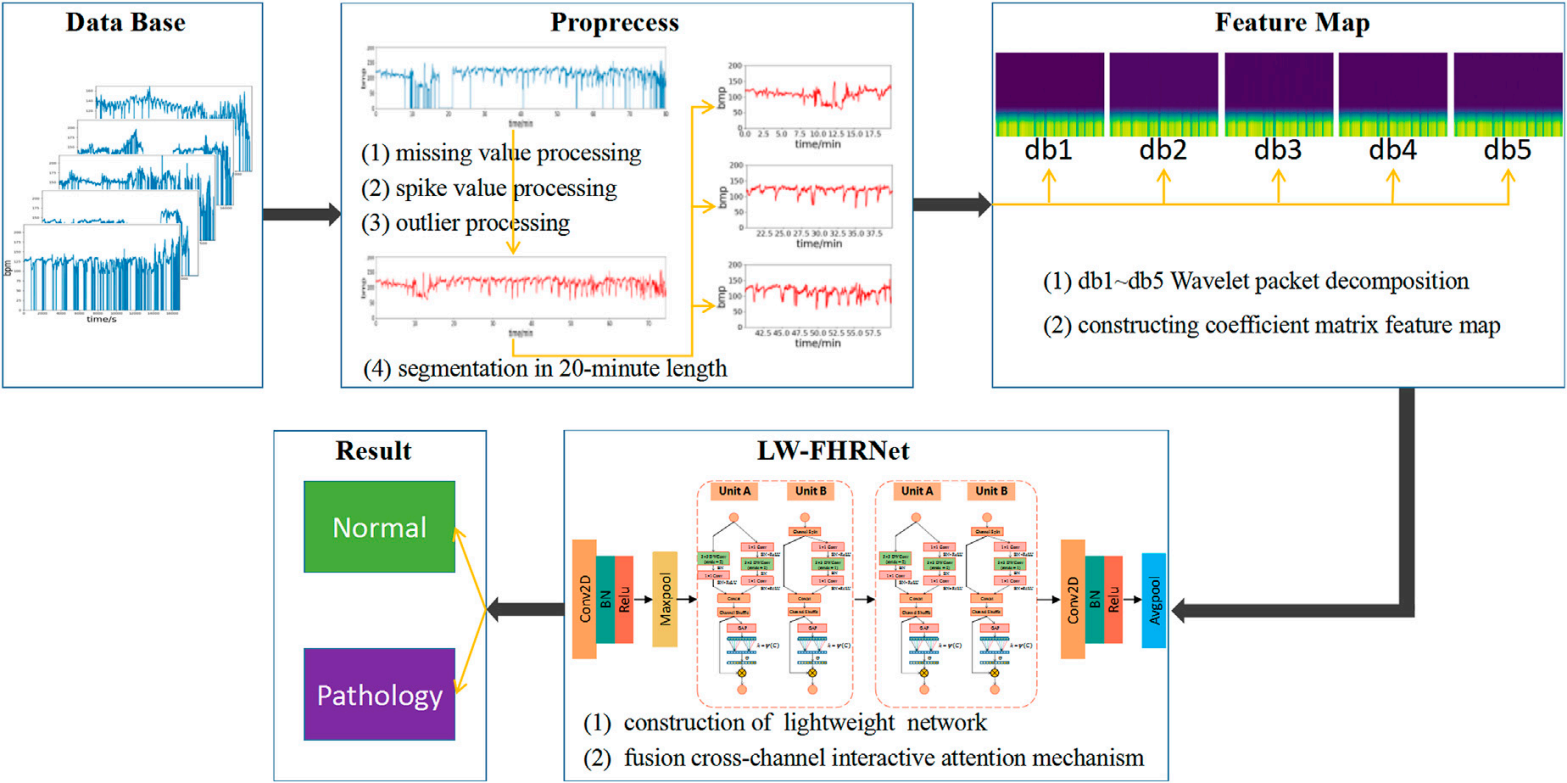
Description of the architecture for the proposed lightweight network-based fetal distress assisted-diagnosis model.

The architecture of the lightweight fetal distress-assisted diagnosis model based on the cross-channel interactive attention mechanism designed in this paper is shown in Figure 1, including a preprocessing module, a feature map construction module, and a feature extraction and classification module. First, the missing values and spikes in the FHR signal are removed by signal preprocessing, and the signal is segmented into 20-min lengths. Second, the wavelet packet decomposition technique is used to construct wavelet coefficient matrix feature maps of FHR signals based on db1 to db5 wavelet basis functions. Finally, LW-FHRNet is constructed by using deep separable convolution, channel shuffle and other techniques and incorporating a local cross-channel interactive attention mechanism without dimensionality reduction, which effectively reduces the number of model parameters and improves the classification accuracy of the model.

### 2.1 Signal preprocessing

Clinically, the FHR signal is acquired mainly by an ultrasound Doppler probe placed in the abdomen of the pregnant woman. During the acquisition process, the signal is inevitably subject to a variety of noise interferences, such as the movement of the fetus and the pregnant woman, improper placement of the sensor and other external factors. The noise of the FHR is represented by spikes (FHR values greater than 200 or less than 50 bpm) and missing values (FHR values equal to 0) (Cesarelli et al., 2007). Accordingly, the purpose of preprocessing is to remove these two types of noise. In this study, the interpolation method is used to remove noise (Chudaek et al., 2009), and the specific process is as follows.

(1) If the FHR value is equal to 0 and the duration is greater than 15 s, the segment is removed directly; otherwise, it is linearly interpolated.
(2) If the FHR value is unstable, i.e., the absolute value of two adjacent points is greater than 25 bpm, and interpolation is performed between the starting sampling point and the first point of the next stabilization segment. A stable segment is defined as five consecutive FHR values where the difference is less than 10 bpm.
(3) If the FHR value is greater than 200 bpm or less than 50 bpm, it is filled in with Hermite spline interpolation.

**FIGURE 2:**
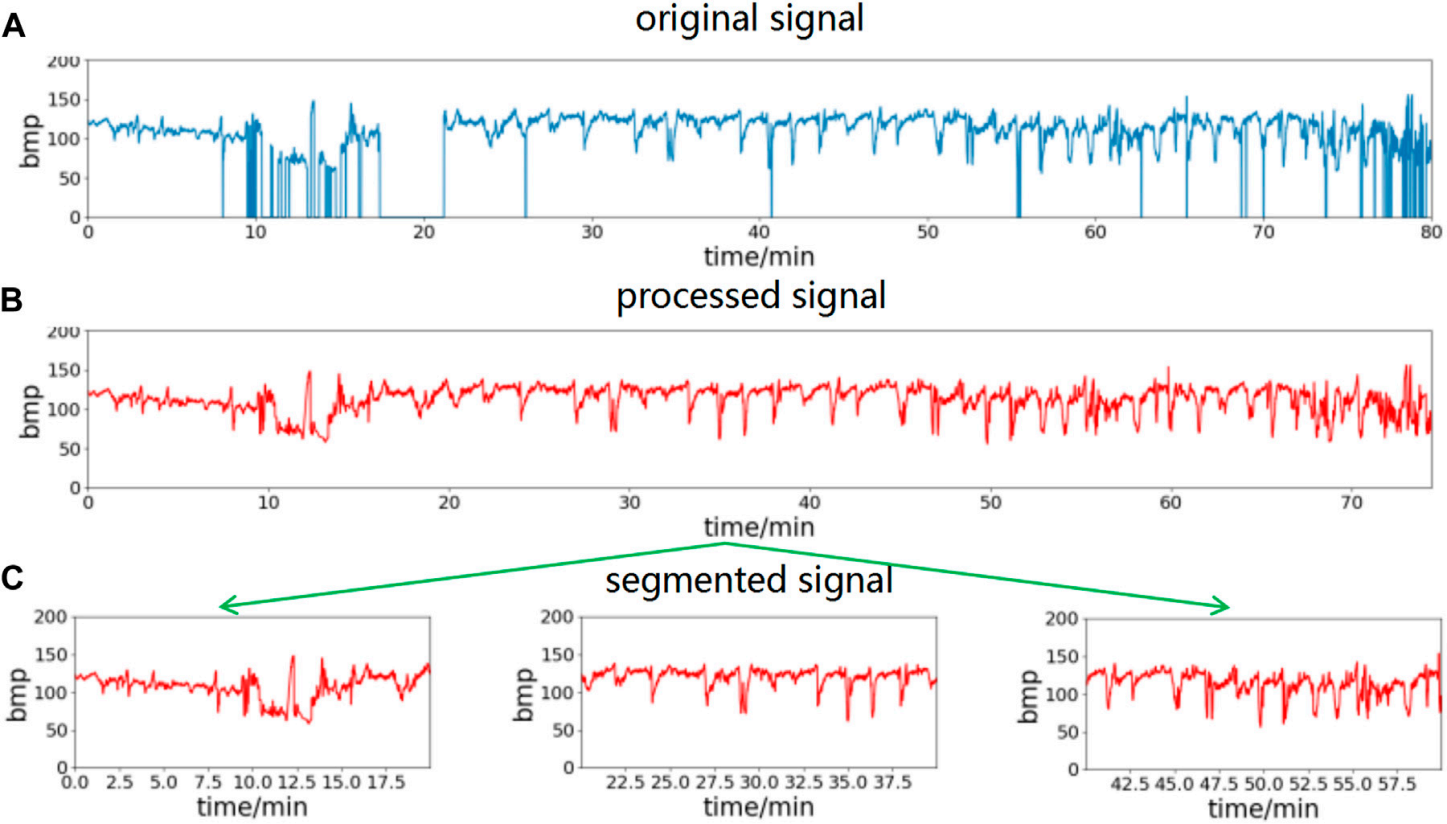
FHR signal preprocessing process. Remove spikes and missing values of the original signal, then divide into segments of 20-min length. **(A)** The original signal, **(B)** processed signal, **(C)** segmented signal.

Noise and missing value segments in the FHR signal can be effectively filtered out by the above interpolation method. In conjunction with the time requirement of clinical prenatal examination, this paper uses 20-min data segments for analysis. The preprocessed data are segmented into 20-min time segments to obtain multicomponent segment data. The waveform obtained using the above preprocessing method is shown in Figure 2, where (a) is the raw data of the FHR signal, (b) is the waveform after preprocessing using the above method, and (c) is the segment after splitting the data into multiple segments with a 20-min data length.

### 2.2 Construction of feature maps based on wavelet packet coefficients

As a non-stationary and non-linear time series, FHR contains complex physiological and pathological information. Wavelet packet decomposition is a discrete analysis method of non-stationary signals that can select the appropriate spectral band according to the signal characteristics and improve the time-frequency analysis resolution (Behera and Jahan, 2012). In this paper, wavelet packet decomposition is introduced to construct the wavelet packet coefficient matrix using different subspace coefficients to convert the 1D FHR signal into a 2D wavelet packet coefficient feature map. The feature map is used as the input layer data for the deep network model.

**FIGURE 3:**
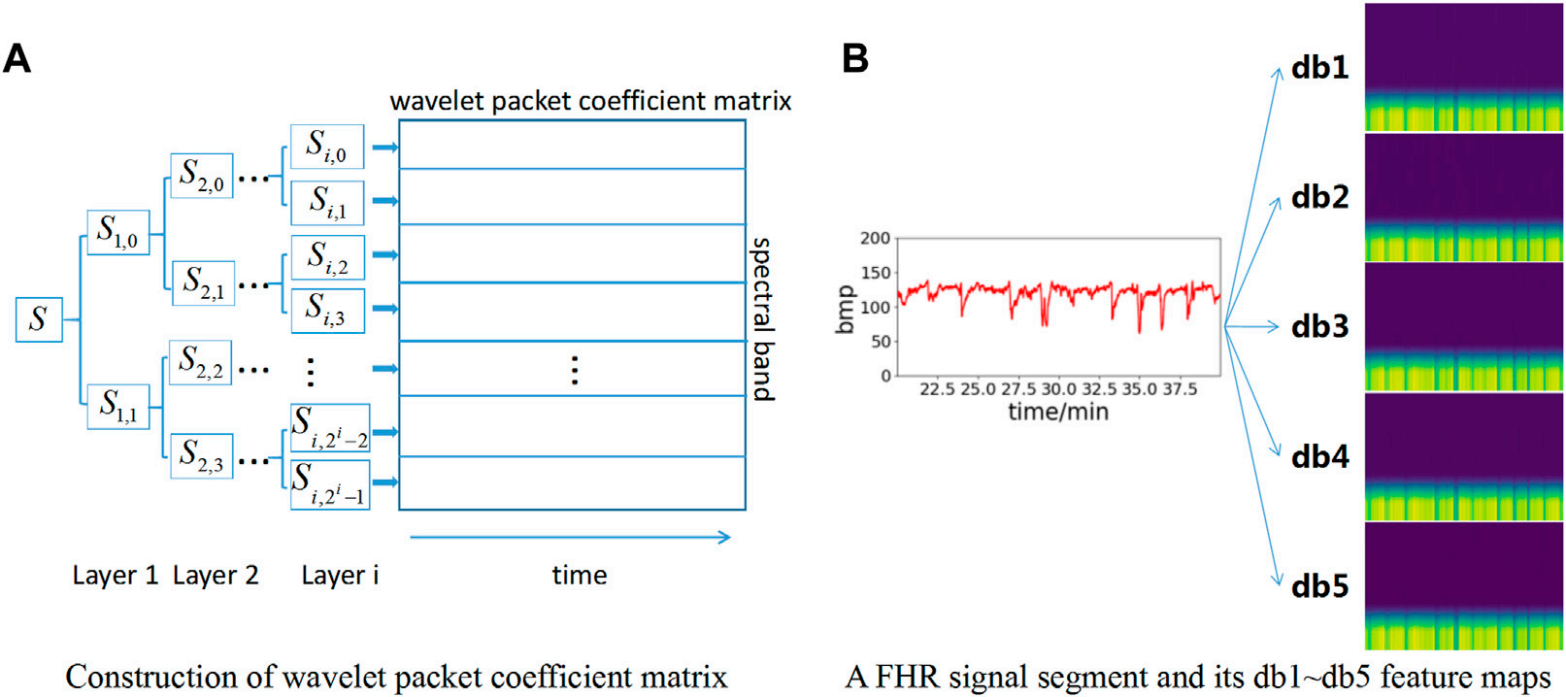
Construction of feature maps based on wavelet packet coefficient matrix. **(A)** Construction of wavelet packet coefficient matrix; **(B)** Construction of db1∼db5 feature map.

Figure 3A shows the wavelet packet coefficient matrix construction process. The signal is decomposed into corresponding frequency bands through different layers, and each frequency band has a series of wavelet packet coefficients. For the *n*th layer decomposition, the wavelet packet transform provides 2^n^ different subspaces, and each subspace corresponds to a frequency band.

Wavelet packet decomposition can be implemented using a series of convolutions with high-pass filters and low-pass filters. The high-pass filter $h\left(\cdot \right)$ and low-pass filter $g\left(\cdot \right)$ can be defined as Eqs 1, 2.

$$h\left(k\right)=\frac{1}{\sqrt{2}}\langle \varphi \left(t\right),\varphi \left(2t-k\right)\rangle$$ (1)

$$g\left(k\right)=\frac{1}{\sqrt{2}}\langle \psi \left(t\right),\psi \left(2t-k\right)\rangle$$ (2)

where $\phi \left(t\right)$ is the scale function, $\psi \left(t\right)$ is the wavelet function, $\langle \cdot ,\cdot \rangle$ represents the inner product, and t and k are variables. $h\left(\cdot \right)$ and $g\left(\cdot \right)$ satisfy Eq. 3.

$$g\left(k\right)={\left(-1\right)}^{k}h\left(1-k\right)$$ (3)

The wavelet coefficients at different frequency bands and decomposition layers can be calculated iteratively by the following equation.

$$S_{i+1,2j}\left(\tau \right)=\underset{k}{\sum }h\left(k-2\tau \right)S_{i,j}\left(k\right)$$ (4)

$$S_{i+1,2j+1}\left(\tau \right)=\underset{k}{\sum }g\left(k-2\tau \right)S_{i,j}\left(k\right)$$ (5)

where $S_{0,0}$ is the original signal of length N, $\left\{S_{i,j}\left(k\right),k=1,2,...,N/2^{i}\right\}$ are the wavelet coefficients in the *j*th subfrequency band at the *i*th layer decomposition, $\left\{S_{i+1,2j}\left(\tau \right),\tau =1,2,...,N/2^{i+1}\right\}$ and $\left\{S_{i+1,2j+1}\left(\tau \right),\tau =1,2,...,N/2^{i+1}\right\}$ are the wavelet coefficients in the (2j)-th and (2j+1)-th subfrequency bands at the (i+1)-th layer decomposition, and for the *i*th layer decomposition $j\in \left\{0,1,...,2^{i}-1\right\}$ .

To increase the number of datasets to obtain better model effects, db1∼db5 wavelet basis functions are selected for wavelet packet coefficient decomposition in this paper. Therefore, five wavelet packet coefficient matrix maps can be obtained for each data segment to enhance the dataset. Meanwhile, each wavelet packet matrix coefficient map is resized to 224*224*3 pixels as the input layer of the neural network model. The feature map construction based on wavelet packet coefficients is shown in Figure 3B. Each FHR signal segment is converted into a total of 5 feature maps based on db1∼db5 wavelet bases.

### 2.3 LW-FHRNet network structure

To meet the application of deep neural networks on embedded and mobile terminals and maintain excellent performance, lightweight network models have emerged. In particular, the lightweight models of the MobileNet series and the ShuffleNet series are the most widely used. Depthwise separable convolution, pointwise convolution, group convolution, channel shuffle and channel separation are used to reduce the number of model parameters and speed up the model computation time.

Recently, the channel attention mechanism has been shown to have great potential in improving the performance of deep convolutional neural networks. By assigning different weights to each part of the input, more important information can be extracted to help the model make more accurate judgments without imposing greater overhead on the model’s computation and storage.

**FIGURE 4:**
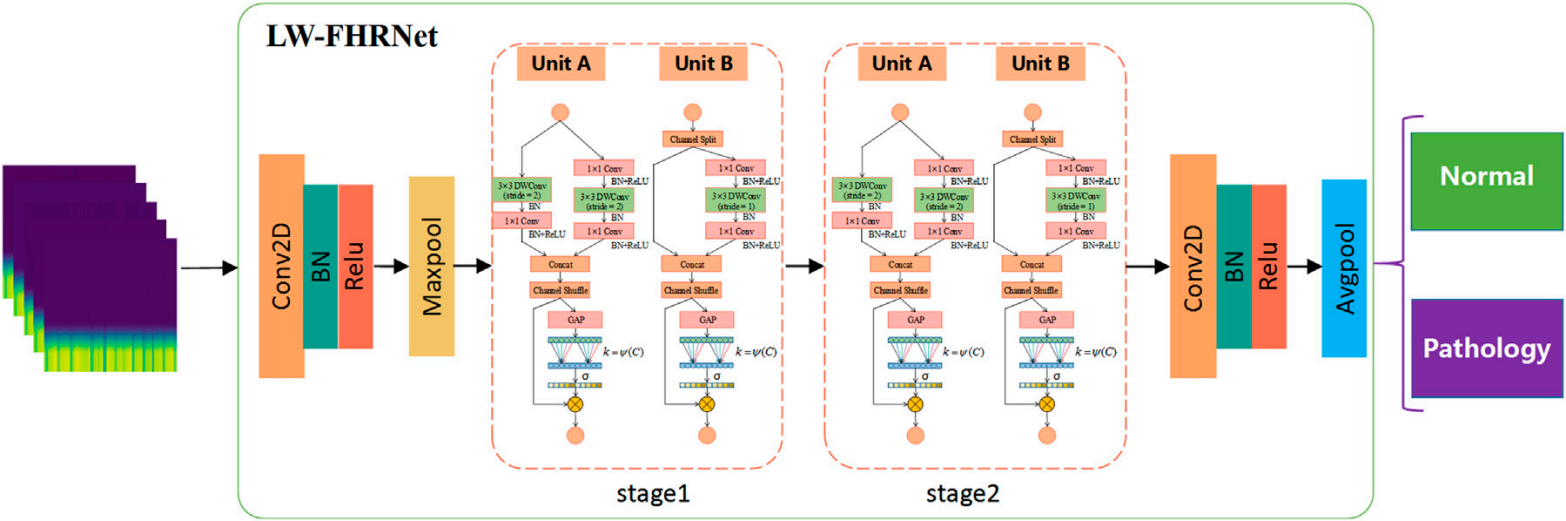
The structure of LW-FHRNet. Notes: Conv2D: Convolution2D; BN: Batch Normalization; Maxpool: Max pooling; Avgpool: Average pooling.

Inspired by the above work, a lightweight network based on a cross-channel attention mechanism, LW-FHRNet, is proposed in this work to assist in the diagnosis of fetal distress symptoms, as shown in Figure 4. The main structure of the network contains two stages and a total of four ECA-Shuffle units. First, the feature maps based on wavelet packet coefficients are used as the input layer of the model. Subsequently, the image is conventionally convolved and the size of the output feature matrix is reduced to 1/4 of the input image using the maximum pooling operation. Then, feature extraction is performed by 4 ECA-Shuffle units to fully learn the feature unit information. Finally, regular convolution and average pooling are performed, and the output features are sent to the fully connected layer for classification.

**FIGURE 5:**
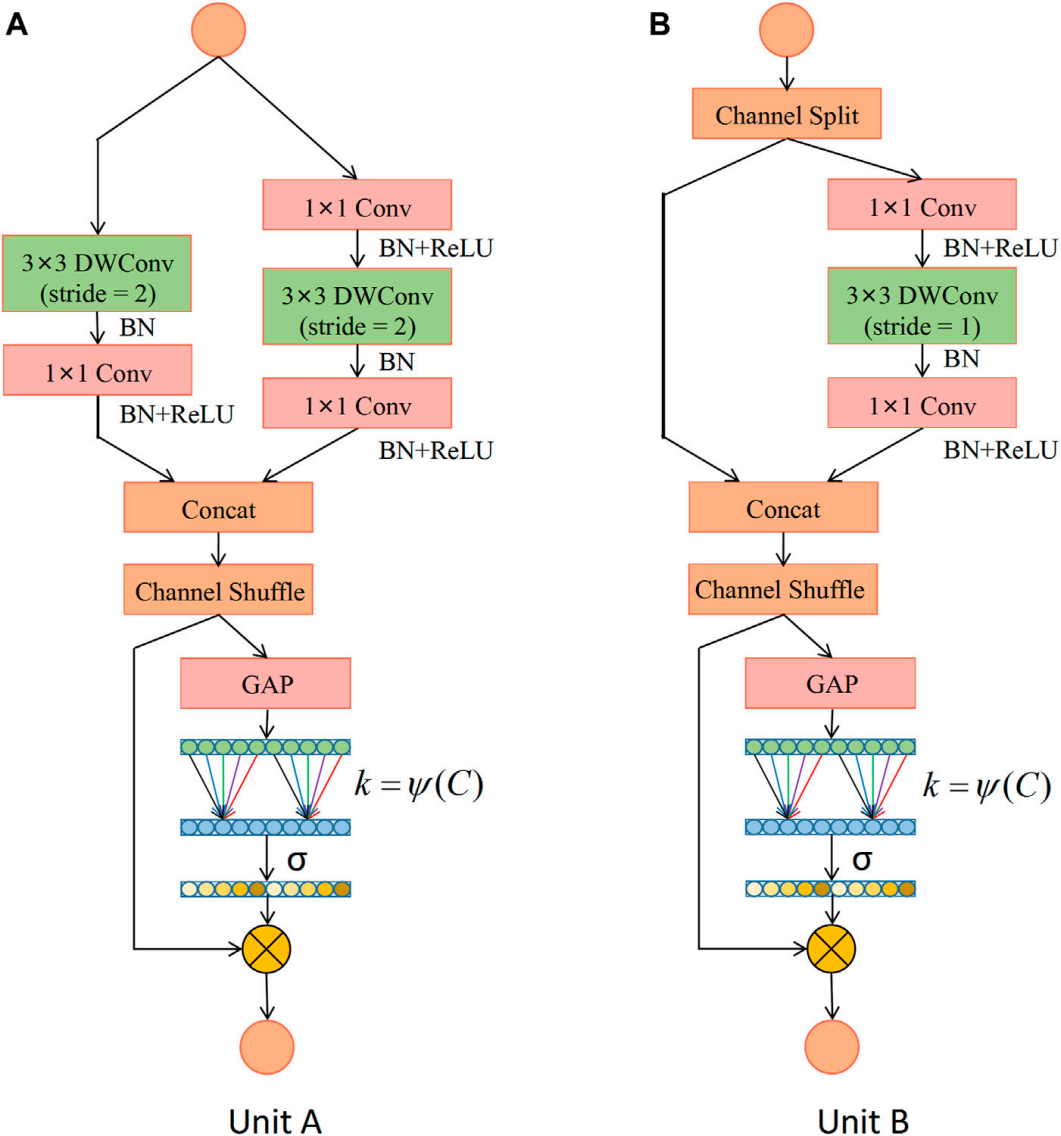
Detailed description of the ECA-Shuffle unit. **(A)** Unit A: the basic unit for spatial down sampling; **(B)** Unit B: the basic unit for channel split. Notes: DWConv: Depthwise separable convolution; Conv: convolution; BN: Batch Normalization; GAP: Global Average Pooling.

Based on the ShuffleNet-V2 units, this study constructs two types of ECA-Shuffle units by integrating the cross-channel attention module without dimensionality reduction, as shown in Figure 5. Figure 5A (Unit A) shows the first unit of each stage. The stride of the depthwise separable convolution in both the residual branch and the identity branch of the bottleneck structure is 2, and the two output feature matrices are concatenated to 2 times their depth. The ECA strategy is used at the tail of the structure. Figure 5B (Unit B) shows the second unit of each stage. The input feature matrix is divided equally into two groups. The main branch performs a depthwise separable convolution with a stride of 1, while the other branch is left unprocessed and connected to the main branch *via* concat, and the feature matrix depth is kept constant. The ECA strategy is also used at the end of the structure.

**FIGURE 6:**
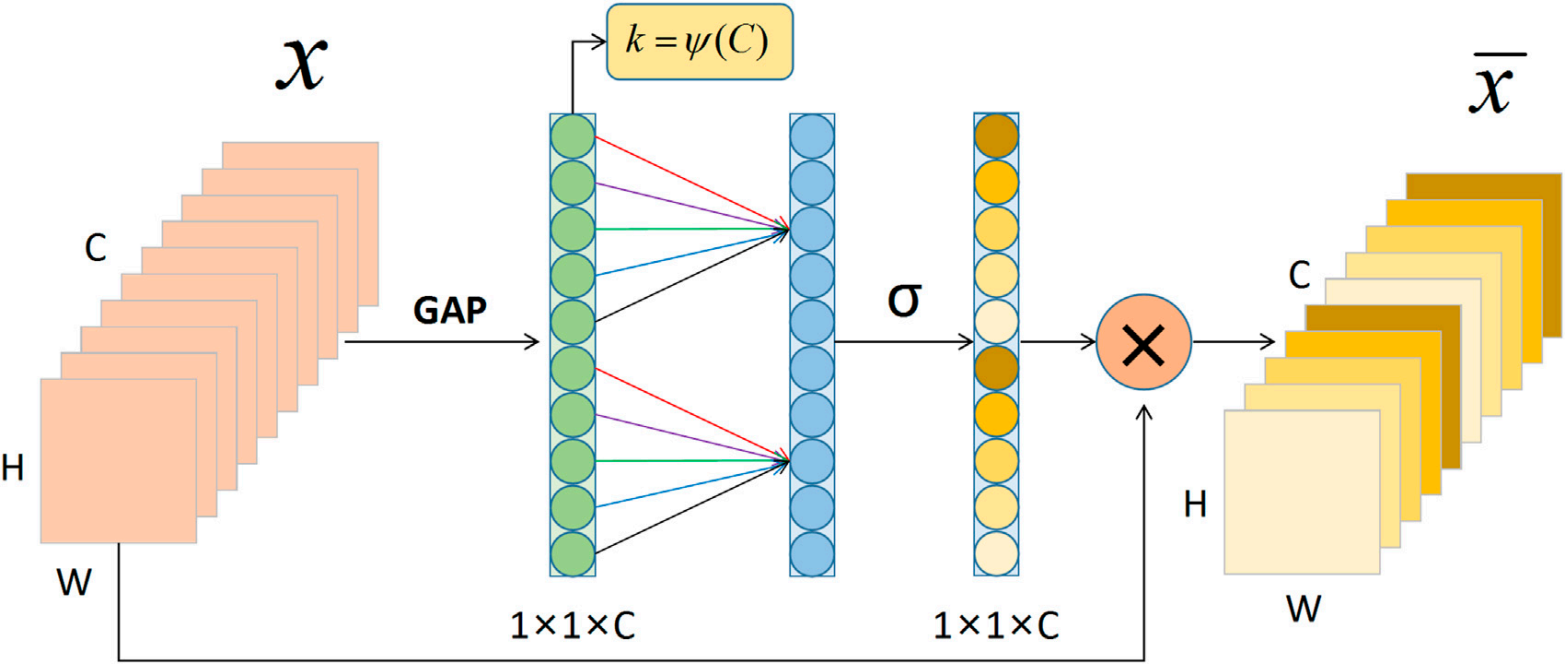
The cross-channel interactive attention module. Notes: GAP: Global Average Pooling; C: Channel dimension; H: Height; W: Width.

The lower half of the ECA-Shuffle unit is the cross-channel interactive attention module without dimensionality reduction. The detailed structure is shown in Figure 6. Given the aggregated feature $y\in R^{C}$ without dimensionality reduction, channel attention can be learned by Eq. 6.

ω=σ(Wy) (6)

If the weight of $y_{i}$ is calculated by only considering the interaction between $y_{i}$ and its k neighbors and all channels share the same learning parameters, Eq. 6 can be written as Eq. 7.

$$\omega_{i}=\sigma \left(\overset{k}{\underset{j=1}{\sum }}w^{j}y_{i}^{j}\right),y_{i}^{j}\in \Omega_{i}^{k}$$ (7)

where $\Omega_{i}^{k}$ indicates the set of k adjacent channels of $y_{i}$ . This strategy can be easily implemented by a fast 1D convolution with kernel size k , i.e.,

$$\omega =\sigma \left(C1D_{k}\left(y\right)\right)$$ (8)

where C1D denotes 1D convolution.

Considering each channel and its k nearest neighbors, computing local cross-channel interaction information instead of all channels effectively improves computational efficiency. This efficient channel attention calculation can be quickly implemented by 1D convolution. Thus, k is the key parameter and the size of the convolution kernel of the 1D convolution, which determines the range and convergence of the local cross-channel interaction.

To avoid resource-consuming cross-validation adjustment, an adaptive method is used to select the appropriate k value. According to the properties of group convolution, the high-dimensional (low-dimensional) channels are proportional to the long-distance (short-distance) convolution for a fixed number of groups. Similarly, the coverage of the interaction (i.e., the size k of the 1D convolution kernel) is proportional to the channel dimension C . The mapping relationship between k and C is shown in Eq. 9.

$$C=\phi \left(k\right)$$ (9)

Since the channel dimension is generally an exponential multiple of 2, the non-linear mapping relationship is represented by an exponential function with a base of 2. Thus, Eq. 9 can be rewritten as Eq. 10.

$$C=\phi \left(k\right)=2^{\left(\gamma *k-b\right)}$$ (10)

Consequently, the size k of the convolution kernel can be calculated automatically based on the number of channels C , which is given by Eq. 11.

$$k=\psi \left(C\right)={\left|\frac{\log_{2}\left(C\right)}{\gamma }+\frac{b}{\gamma }\right|}_{odd}$$ (11)

where ${\left|t\right|}_{odd}$ represents the nearest odd number of t . To reduce the computational cost and training time, γ and b are empirically set to 2 and 1, respectively.

The details of the lightweight network: LW-FHRNet structure designed in this work are shown in Table 1. The first operation of each stage is the ECA-Shuffle unit A, which realizes the doubling of feature dimensions, followed by the ECA-Shuffle unit B, which realizes the subsequent operations.

**TABLE 1:** The structure parameter information of LW-FHRNet.

Layer	Output size	Kernel size	Output channel
Input	224 × 224	-	3
Conv	112 × 112	3 × 3	24
MaxPool	56 × 56	3 × 3	
Stage1	28 × 28	-	116
Stage2	14 × 14	-	232
Conv	14 × 14	1 × 1	1024
AvgPool	1 × 1	14 × 14	
FC	-	-	1

The normalization and ReLU, layers that follow each convolutional layer are not shown above because they do not change the output feature shape. Conv: convolutional layer; MaxPool: max pooling layer; AvgPool: average pooling layer; FC: fully connect layer; stage: ECA-Shuffle uint A+ ECA-Shuffle uint B.

The process of the fetal distress classification algorithm based on a lightweight network is described in Table 2. After preprocessing and 20-min length segmentation, the dataset is randomly divided into a training set and a testing set in proportion. Each segment is subjected to wavelet packet decomposition based on db1 to db5 wavelet basis functions to obtain five feature maps. Iterative testing of model tuning is performed with the training set data to obtain the optimal model. The testing set is subjected to category prediction under the optimal model, and the final category attribution is decided by voting on the five feature maps of each data segment.

**TABLE 2:** Details of LW-FHRNet classification algorithm.

Input: $S^{train}$ training sample sets; $L^{train}$ training label sets, $S^{test}$ testing sample sets; $L^{test}$ testing label sets Output: Prediction label ${\tilde{L}}^{test}$ of the $S^{test}$
1: **for** dbi in $\left[db1,db2,db3,db4,db5\right]$ **do** 2: $F_{dbi}^{train}=PWT_{dbi}\left(S^{train}\right)$ # $PWT_{dbi}\left(\cdot \right)$ is the wavelet packet decomposition based on the dbi wavelet basis functions 3: $L_{dbi}^{train}=L^{train}$ 4: $F_{dbi}^{test}=PWT_{dbi}\left(S^{test}\right)$ 5: $L_{dbi}^{test}=L^{test}$ 6: **end for**
7: # training procedure 8: Initialize parameters and weights 9: **for** i in [1, 2, 3, 4, 5] **do** 10: metrics = $LW-FHRNet\left(F_{dbi}^{train},L_{dbi}^{train}\right)$ 11: Train the LW-FHRNet model by optimizing the loss function 12: **end for** 13: **return** model LW-FHRNet-best
14: # testing procedure 15: **for** i in [1, 2, 3, 4, 5] **do** 16: $L_{dbi}^{test}\overset{predict}{\leftarrow }$ LW-FHRNet-best ( $F_{dbi}^{test}$ ) 17: **end for** 16: ${\tilde{L}}^{test}=vote\left(L_{db1}^{test},L_{db2}^{test},L_{db3}^{test},L_{db4}^{test},L_{db5}^{test}\right)$ #Vote () s a voting function 17: **return** ${\tilde{L}}^{test}$

## 3 Results

### 3.1 Dataset

The database in this paper uses the publicly available dataset CTU-UHB, which comes from the Czech Technical University in Prague (CTU) and the University Hospital in Brno (UHB) (Chudacek et al., 2014). A total of 552 CTG records were collected in the database. These records were carefully selected from 9,164 records collected by UHB from 2010 to 2012. The sampling rate of CTG data is 4 Hz, and each CTG record contains FHR sequences and UC sequences. The records in the database were all singleton gestations, all gestational ages greater than 36 weeks and no known congenital developmental defects. The quality of the FHR signal was greater than 50% in every 30-min window. Available biochemical parameters of the umbilical artery blood sample (pH) were recorded for each sample.

The pH value is a marker of blood acid-base balance and can provide information on possible fetal acidosis caused by intrauterine hypoxia. A lower pH value represents a more severe degree of fetal acidosis (Vayssiere et al., 2007). showed moderate ability to detect mild acidosis at pH ≤ 7.15 and better ability to detect more severe acidosis at pH ≤ 7.05. Therefore, in this paper, pH = 7.05 was chosen as the criterion to classify the data into two categories. Data with a pH value greater than 7.05 are considered normal, and data with a pH value less than or equal to 7.05 are considered abnormal. Based on this discriminant, 44 abnormal samples and 508 normal samples are obtained (Ito et al., 2022). predicted fetal acidemia by calculating iPREFACE (10), iPREFACE (30) and iPREFACE (60) at 10, 30, and 60 min before delivery. The results showed that iPREFACE (30) was slightly better than iPREFACE (60) but significantly better than iPREFACE (10). To enhance the sample size, a 20-min segmentation is performed after preprocessing the 60-min data before delivery. After splitting the samples into 20-min data segments, 106 abnormal sample segments are obtained. To avoid the effect of overfitting or underfitting caused by category imbalance on the classification results, 106 samples from 512 normal samples are randomly selected. The second 20-min segment is selected to construct 106 normal sample segments for the experiment. Eighty percent of the dataset is randomly selected as the training set (85P and 85N), and the remaining 20% as the test set (21P and 21N). The wavelet packet decomposition from the db1 to db5 wavelet basis is performed separately for each FHR data segment, which constitutes 5 wavelet packet coefficient matrix feature maps. Therefore, there are 850 images in the training set and 210 images in the test set.

**FIGURE 7:**
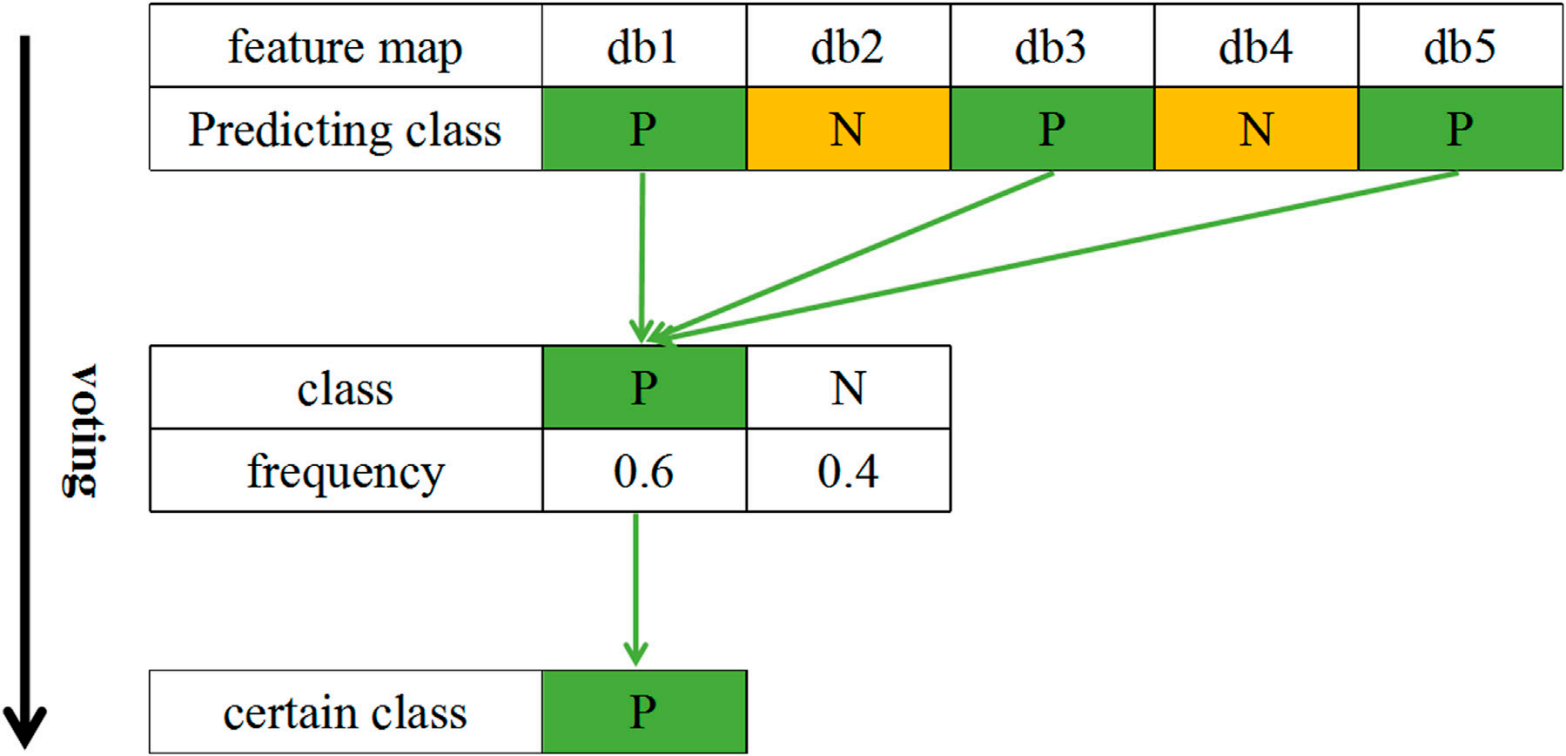
An example of the category voting process. Notes: P: Positive; N: Negative.

In this paper, each 20-min segment of FHR data is subjected to wavelet packet decomposition based on db1 to db5 wavelet basis functions to obtain five wavelet coefficient matrix feature maps. Category attribution is determined by voting on the 5 feature maps. The category voting process is shown in Figure 7. First, each feature map of the segment is classified. Subsequently, the frequency of each category label is calculated for the segment. Finally, the class with higher frequency is selected as the category of this FHR segment.

### 3.2 Experimental setup

#### 3.2.1 Environment

The network structure proposed in this paper is trained and tested on the CTU-UHB dataset. The experimental platform is a computer equipped with an Intel Xeon(R) CPU E3-1535M v6 @ 3.10 GHz x 8, Quadro P5000 GPU and 32 G RAM. The system is Ubuntu 18.04.6LTS, the development environment is TensorFlow 2.6.2, and the language used is Python.

#### 3.2.2 Metrics

To evaluate the classification performance of the model, accuracy, precision, recall and F1-Score metrics are used in this paper. Additionally, model parameters and model size are introduced to evaluate the complexity of lightweight models. Finally, sensitivity (Se) and specificity (Sp) are used to observe the discriminatory ability of the model between abnormal and normal samples.

#### 3.2.3 Baselines

The commonly used lightweight networks MobileNetV3-Small, MobileNetV3-Large and ShuffleNet-V2 are introduced as the baselines of this research. MobileNetV3 introduces the channel attention module based on MobileNetV2 to enhance the adaptive capability of the model by assigning different weights to different channels. MobileNetV3 has two versions: small and large. ShuffleNet-V2 proposes the concept of channel separation to replace group convolution to further improve the inference speed.

### 3.3 Experiment 1: Selection of wavelet packet decomposition layers

Wavelet packet decomposition with different numbers of layers can obtain different detailed information. The sampling frequency of the raw data is 4 Hz. The *i*th layer is decomposed to obtain 2^i^ frequency bands. The 2D image is constructed according to the frequency from the highest to the lowest. The frequency range of the *j*th frequency band is $\left(\frac{4}{2^{i}}\left(j-1\right)∼\frac{4}{2^{i}}j\right)$ Hz, $j\in \left[1,2^{i}\right]$ 。 To select the best wavelet coefficient matrix feature map, this paper performs wavelet packet 1-layer to 5-layer decomposition to obtain the wavelet packet coefficient matrix maps of corresponding layers to test the classification performance. The experimental results are shown in Table 3. The accuracy of the 2-layer and 3-layer decomposition is higher, and the accuracy of the 4-layer and 5-layer decomposition gradually decreases. The 2-layer decomposition achieves optimal performance with 95.24% accuracy, 100% precision, 90.48% recall and a 95.00% F1-score. Therefore, the feature map based on 2-layer wavelet packet decomposition is chosen as the input of the model in this paper. That is, the signal is decomposed into four frequency bands:0–1 Hz, 1–2 Hz, 2–3 Hz and 3–4 Hz. And the wavelet packet coefficients in the corresponding frequency bands are used to jointly construct the feature maps.

**TABLE 3:** Performance comparison of feature maps constructed by different layers of wavelet packet decomposition.

Decomposition Level	Accuracy (%)	Precision (%)	Recall (%)	F1-score (%)
layer 1	83.33	85.00	80.95	82.93
**layer 2**	**95.24**	**100**	**90.48**	**95.00**
layer 3	90.48	94.74	85.71	90.00
layer 4	80.95	84.21	76.19	80.00
layer 5	76.19	76.19	76.19	76.19

The bold values means the best performance.

### 3.4 Experiment 2: The effective role of local cross-channel interactive attention mechanisms

**FIGURE 8:**
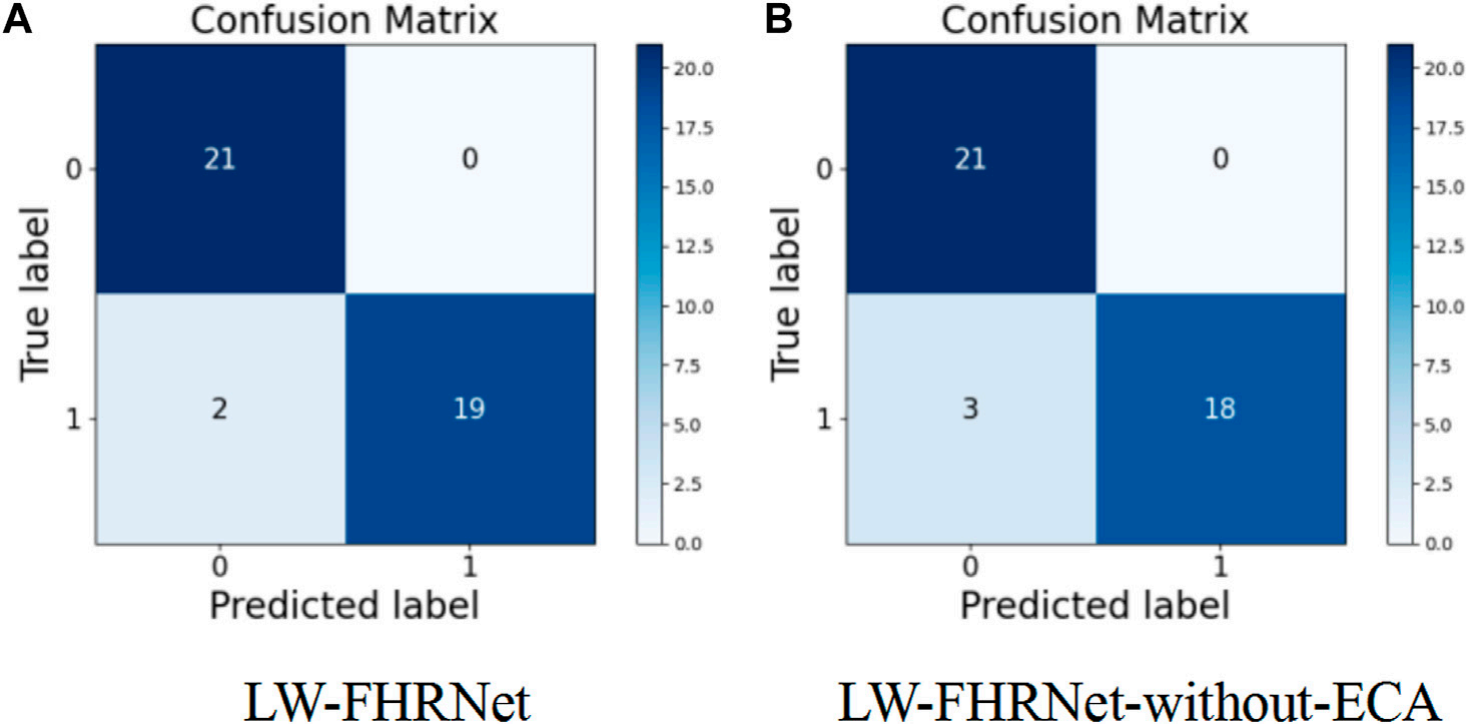
Confusion matrix. **(A)** The proposed LW-FHRNet, **(B)** the proposed LW-FHRNet without the ECA module.

The channel attention mechanism has great potential to improve the performance of deep convolutional neural networks. In this paper, we introduce a cross-channel local interaction attention strategy without dimensionality reduction to improve the performance of lightweight models. Experiments are conducted on the dataset of this paper using a lightweight network with and without an ECA module. The confusion matrix of whether the proposed lightweight model contains ECA modules is shown in Figure 8. Table 4 shows the model performance comparison with and without the ECA module. The lightweight model accuracy with the ECA module is as high as 95.24%, and the accuracy of the lightweight model without the ECA module is 92.86%. The experimental results show that the lightweight model with the ECA module improves performance in fetal distress classification.

**TABLE 4:** Lightweight model performance comparison with and without the ECA module.

Model (%)	Accuracy (%)	Precision (%)	Recall (%)	F1 score (%)
**LW-FHRNet**	**95.24**	**100**	**90.48**	**95.00**
LW-FHRNet-without-eca	92.86	100	85.71	92.31

The bold values means the best performance.

### 3.5 Experiment 3: Lightweight model comparison experiment

To clarify the performance of the network, this paper performs a comparative test with different lightweight networks. The classification performance of fetal distress under different lightweight networks is measured using accuracy, precision, recall, F1-score and model size metrics. The test performance comparison of the LW-FHRNet network with other commonly used lightweight networks is shown in Table 5. MobileNetV3 improves MobileNetV2 by using a deep separable convolution +SE channel attention mechanism + residual structure connection to further reduce the computational effort. The overall structure of small and large is the same, and the difference is the number of bnecks and channels. MobileNetV3-Small achieves 85.71% accuracy, proving that the network has a strong feature learning capability. MobileNetV3-Large has better accuracy than MobileNetV3-Small, but the number of network parameters has increased significantly due to the increase in the number of bnecks and channels. The ShuffleNet-V2 network improves the ShuffleNet-V1 network architecture in terms of optimizing memory access cost (MAC), reducing network fragmentation, and decreasing element operations. Due to the small number of parameters in the ShuffleNet-V2 model, it performs poorly in terms of accuracy, with only 83.33%. Due to the low number of parameters in the ShuffleNet-V2 model, its performance is relatively poor, with an accuracy of 83.33%.

**TABLE 5:** Performance comparison of different lightweight models for fetal distress classification.

Network	Accuracy (%)		Precision (%)		Recall (%)		F1 score (%)		Parameter (M)			Model size (M)
MobileNetV3-Small	85.71		82.61		90.48		86.36		1.53			5.84
MobileNetV3-Large	90.48		94.74		85.71		90.00		4.23			16.13
ShuffleNet-V2	83.33		85.00		80.95		82.93		1.27			4.85
**LW-FHRNet(ours)**	**95.24**		**100**		**90.48**		**95.00**		**0.33**			**1.27**

The bold values means the best performance.

**FIGURE 9:**
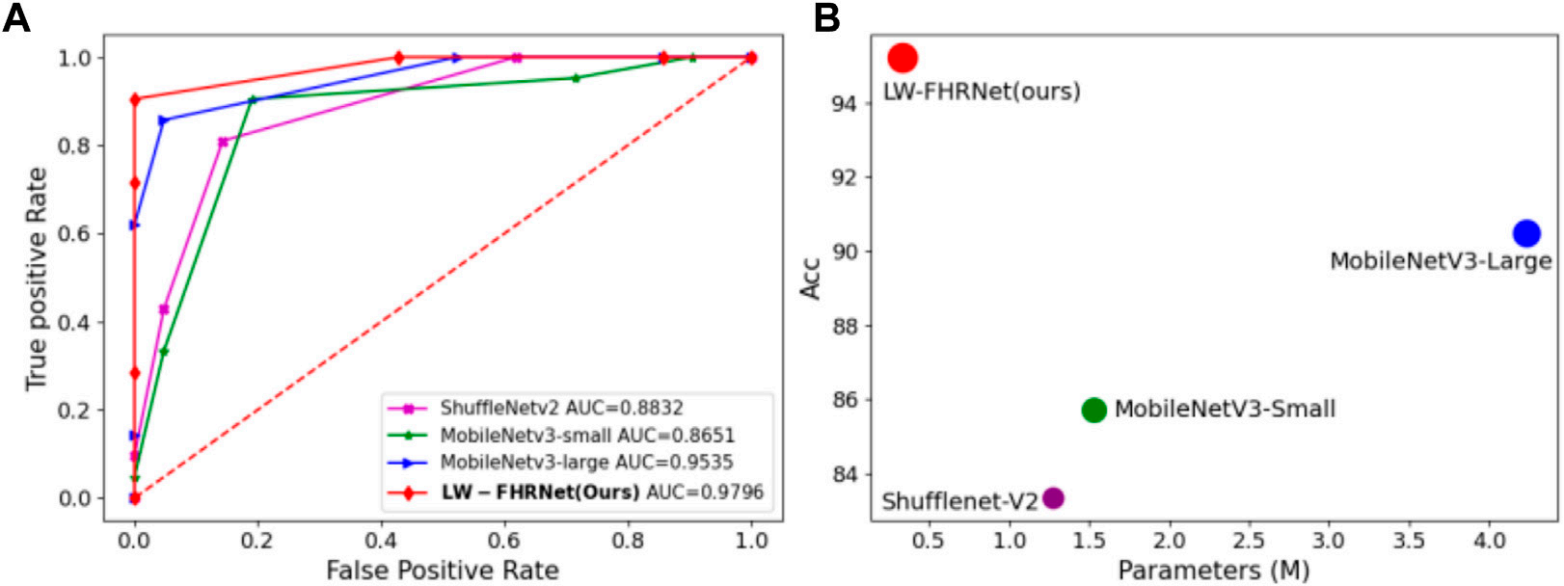
Classification performance of different lightweight models. **(A)** ROC curves of different lightweight models; **(B)** Acc and parameters of different lightweight models, where green, blue, purple, and red refer to MobileNetV3-Small, MobileNetV3-Lagre, ShuffleNetV2, and LW-FHRNet (Ours).

LW-FHRNet incorporates an efficient cross-channel attention mechanism without downscaling on the base unit of ShuffleNet-V2. The channel interaction strategy effectively improves the performance of channel attention and enables LW-FHRNet to have a more accurate recognition performance. The ROC curves of LW-FHRNet and other commonly used lightweight network models are shown in Figure 9A. The proposed network in this paper has the best performance with 97.96% AUC. A comparison of the accuracy and model size of LW-FHRNet with other commonly used lightweight networks for fetal distress classification is shown in Figure 9B. LW-FHRNet achieves 95.24% accuracy for fetal distress classification, which is higher than other commonly used lightweight networks. Additionally, it has the lowest computational cost, and the number of network parameters is only 0.33 M, which is much lower than other commonly used lightweight networks.

## 4 Discussion

In this paper, a lightweight network based on cross-channel interactive attention mechanism is proposed to effectively fuse channel features and reduce model complexity to help obstetricians to objectively assess fetal distress. In the experiments, the classification effects of wavelet packet decomposition with different layers as feature maps were first compared. And the optimal number of wavelet packet decomposition layers was chosen as 2-layer. Then two different network architectures (LW-FHRNet and LW-FHRNet-without-eca) were used. The results showed that the attention machine module effectively improves the classification performance of fetal distress. Finally, a comparison with other lightweight models was made to show that the lightweight network proposed in this paper outperforms other common lightweight networks.

To analyze the significance of the results, the algorithm in this paper is compared with recent related work in the diagnosis of fetal distress using the CTU-UHB database. The results are shown in Table 6, which measures the performance of this research work in terms of accuracy (Acc), sensitivity (Se) and specificity (Sp). Compared with (Zarmehri et al., 2019), the method of this paper has higher Se and Sp under the same fetal distress division criteria, which further highlights the advantages of our model. Compared with (Alsaggaf et al., 2020), they also have good classification accuracy, but they use the traditional machine learning classification method, which requires manual design to extract a large number of features. The feature extraction process is complex and computationally intensive. Compared with (Baghel et al., 2022), they have higher accuracy than the model in this paper, but they use regular CNN convolution for feature extraction. The parameter number and computational time still need to be improved and optimized for end-application deployment.

**TABLE 6:** Comparison of recent studies on the prediction of fetal distress using the CTU-UHB database.

Author	Division criteria	Method	Performance (%)		
			Acc	Se	Sp
Comert and Kocamaz. (2018)	pH ≤ 7.15	BFS, DWT + SVM	67.00	57.42	70.11
Fuentealba et al. (2019)	PH<7.05; BDecf≥12pH>7.20; BDecf≥12	CEEMDAN, TV-AR + SVM	81.7	79.5	86.45
Zarmehri et al. (2019)	pH ≤ 7.05	FFT	—	63.60	80.10
Alsaggaf et al. (2020)	pH < 7.15	Morphological, linear, non-linear, CSP + SVM	94.75	74.29	99.55
Zeng et al. (2021)	pH ≤ 7.05; BE ≤ −10	CWT, WTC, XWT + ECSVM	67.2	85.2	66.1
Liu et al. (2021)	pH ≤ 7.15	CNN-BiLSTM + Attention, DWT	71.71	75.23	70.82
Baghel et al. (2022)	pH ≤ 7.15	1D CNN	99.09	—	—
**Ours**	**pH ≤ 7.05**	**WPT + LW-FHRNet**	**95.24**	**90.48**	**100**

BFS: basic feature set; DWT: discrete wavelet transform; CEEMDAN: complete ensemble empirical mode decomposition with adaptive noise; TV-AR: time-varying autoregressive; CSP: common spatial pattern; CWT: continuous wavelet transform; WTC: wavelet coherence; XWT: Cross-wavelet Transform; ECSVM: ensemble cost sensitive SVM; WPT: wavelet packet transform; Acc: Accuracy; Se: Sensitivity; Sp: Specificity.

The bold values means the best performance.

In conclusion, the lightweight network model based on the cross-channel interactive attention mechanism proposed in this paper achieves better classification results in fetal distress diagnosis. The ShuffleNet-V2 unit combined with the local cross-channel interactive attention mechanism is used to build a lightweight network, which ensures a low number of parameters and achieves effective network performance improvement.

However, one limitation of the study in this paper is the criteria for discriminating between normal and distressed samples. The current work generally endorses the use of umbilical artery blood pH as a criterion for classification, since pH is an objective response to the fetal oxygen cell supply (Zarmehri et al., 2019) and also to the severity of fetal acidosis (Vayssiere et al., 2007). However, as shown in Table 6, a variety of pH values were used in different research works. There is not yet a universally accepted pH value. In future research work, the study will focus on exploring the pH value of pathological samples. Meanwhile, the BDecf index can reflect the degree of fetal acidosis (Liu et al., 2021). Therefore, a more precise classification of fetal distress can be performed by combining pH and BDecf in subsequent studies.

## 5 Conclusion

In this work, a lightweight network (LW-FHRNet) based on ECA-Shuffle units is proposed for fetal distress classification of FHR signals. After preprocessing, the FHR signal is segmented into 20-min segments, and the wavelet packet decomposition operation based on db1 to db5 wavelet basis functions is performed on each segment. Each segment obtains five wavelet packet coefficient matrix feature maps, which are used as input to the model and vote on the classification result. The ECA-Shuffle unit performs feature extraction on the feature map to fully learn the feature information. We integrate an efficient local cross-channel interactive attention mechanism without dimensionality reduction to reduce model complexity and ensure performance improvement. In this paper, the CTU-UHB open source database is used to test the classification performance of the proposed network. A pH value of 7.05 was used as the gold standard for classification. The proposed algorithmic model achieves excellent results of 95.24%, 90.48%, and 100% for Acc, Se and Sp, respectively.

Although the proposed lightweight network achieved good results in classifying fetal distress, there is still a gap to reach the clinical diagnosis level of physicians. In order to achieve better auxiliary diagnosis, we will do further exploration in future work. On the one hand, the data from clinical fetal heart monitoring contain simultaneous UC signals and FHR signals, but only FHR signals are used to assess fetal distress because of the poor quality of UC signals in publicly available datasets. In the clinic, the UC signal is also an important basis for physicians to diagnose fetal distress. Therefore, the combination of FHR signals and UC signals needs to be considered in further studies. On the other hand, we are considering more time-frequency transform features to improve the classification performance for fetal distress, including Empirical Wavelet Transform, Hilbert-Huang Transform, Singular Spectrum Analysis, etc.

## Data Availability

Publicly available datasets were analyzed in this study. This data can be found here: https://www.physionet.org/content/ctu-uhb-ctgdb/1.0.0/.

## References

[B1] Abdulhay E. W. Oweis R. J. Alhaddad A. M. Sublaban F. N. Radwan M. A. Almasaeed H. M. (2014). Review article: Non-invasive fetal heart rate monitoring techniques. Biomed. Sci. Eng. 2 (3), 53–67. 10.12691/bse-2-3-2

[B2] Alsaggaf W. Comert Z. Nour M. Polat K. Brdesee H. Togacar M. (2020). Predicting fetal hypoxia using common spatial pattern and machine learning from cardiotocography signals. Appl. Acoust. 167, 107429. 10.1016/j.apacoust.2020.107429

[B3] Baghel N. Burget R. Dutta M. K. (2022). 1D-FHRNet: Automatic diagnosis of fetal acidosis from fetal heart rate signals. Biomed. Signal Process. Control 71, 102794. 10.1016/j.bspc.2021.102794

[B4] Barquero-Perez O. Santiago-Mozos R. Lillo-Castellano J. M. Garcia-Viruete B. Goya-Esteban R. Caamano A. J. (2017). Fetal heart rate analysis for automatic detection of perinatal hypoxia using normalized compression distance and machine learning. Front. Physiology 8, 113. 10.3389/fphys.2017.00113 PMC5329001 28293198

[B5] Behera B. Jahan Q. (2012). Wavelet packets and wavelet frame packets on local fields of positive characteristic. J. Math. Analysis Appl. 395 (1), 1–14. 10.1016/j.jmaa.2012.02.066

[B6] Bernardes J. Costa-Pereira A. Ayres-de-Campos D. van Geijn H. P. Pereira-Leite L. (1997). Evaluation of interobserver agreement of cardiotocograms. Int. J. Gynaecol. obstetrics official organ Int. Fed. Gynaecol. Obstetrics 57 (1), 33–37. 10.1016/s0020-7292(97)02846-4 9175667

[B7] Blickstein I. Green T. (2007). Umbilical cord blood gases. Clin. Perinatology 34 (3), 451–459. 10.1016/j.clp.2007.05.001 17765493

[B8] Bobrow C. S. Soothill P. W. (1999). Causes and consequences of fetal acidosis. Archives Dis. Child. Fetal neonatal Ed. 80 (3), F246–F249. 10.1136/fn.80.3.F246 PMC1720942 10212094

[B9] Cao Y. Wei T. Zhang B. Lin N. Rodrigues J. J. P. C. Li J. (2021). ML-Net: Multi-Channel lightweight network for detecting myocardial infarction. Ieee J. Biomed. Health Inf. 25 (10), 3721–3731. 10.1109/jbhi.2021.3060433 33606647

[B10] Cesarelli M. Romano M. Bifulco P. Fedele F. Bracale M. (2007). An algorithm for the recovery of fetal heart rate series from CTG data. Comput. Biol. Med. 37 (5), 663–669. 10.1016/j.compbiomed.2006.06.003 16893537

[B11] Chen W. Gao L. Li X. Shen W. (2022). Lightweight convolutional neural network with knowledge distillation for cervical cells classification. Biomed. Signal Process. Control 71, 103177. 10.1016/j.bspc.2021.103177

[B12] Chudacek V. Spilka J. Bursa M. Janku P. Hruban L. Huptych M. (2014). Open access intrapartum CTG database. Bmc Pregnancy Childbirth 14, 16. 10.1186/1471-2393-14-16 24418387 PMC3898997

[B13] Chudaek V. Huptych M. Koucky M. Spilka J. Bauer L. Lhotska L. (2009). “Fetal heart rate data pre-processing and annotation,” in 9th international conference on information technology and applications in biomedicine, ITAB 2009, AGIOS THERISSOS M.R.1 (Larnaka, Cyprus: IEEE). 10.1109/itab.2009.5394441

[B14] Comert Z. Kocamaz A. F. (2018). “Fetal hypoxia detection based on deep convolutional neural network with transfer learning approach,” in 7th computer science on-line conference, CSOC 2018 (Springer, Cham), 239–248.

[B15] Comert Z. Yang Z. Velappan S. Boopathi A. M. Kocamaz A. F. (2018). “Performance evaluation of empirical mode decomposition and discrete wavelet transform for computerized hypoxia detection and prediction,” in 26th IEEE Signal Processing and Communications Applications Conference, SIU, Izmir, Turkey, 02-05 May 2018 (IEEE), 1–4. 10.1109/siu.2018.8404243

[B16] Fuentealba P. Illanes A. Ortmeier F. (2019). Cardiotocographic signal feature extraction through CEEMDAN and time-varying autoregressive spectral-based analysis for fetal welfare assessment. Ieee Access 7, 159754–159772. 10.1109/access.2019.2950798

[B17] Georgoulas G. Karvelis P. Spilka J. Chudacek V. Stylios C. D. Lhotska L. (2017). Investigating pH based evaluation of fetal heart rate (FHR) recordings. Health Technol. 7 (2), 241–254. 10.1007/s12553-017-0201-7 PMC5686283 29201590

[B18] Grivell R. M. Alfirevic Z. Gyte G. M. L. Devane D. (2015). Antenatal cardiotocography for fetal assessment. Cochrane Database Syst. Rev. 9, CD007863. 10.1002/14651858.CD007863.pub4 PMC6510058 26363287

[B19] Howard A. G. Zhu M. Chen B. Kalenichenko D. Wang W. Weyand T. (2017). MobileNets: Efficient convolutional neural networks for mobile vision applications. *arXiv* .

[B20] Howard A. Sandler M. Chen B. Wang W. Chen L. C. Tan M. (2019). Searching for mobileNetV3. Proc. IEEE Int. Conf. Comput. Vis. 2019, 1314–1324. 10.1109/iccv.2019.00140

[B21] Ito A. Hayata E. Nagasaki S. Kotaki H. Shimabukuro M. Sakuma J. (2022). Optimal duration of cardiotocography assessment using the iPREFACE score to predict fetal acidemia. Sci. Rep. 12 (1), 13064. 10.1038/s41598-022-17364-z 35906383 PMC9338067

[B22] Liu M. Lu Y. Long S. Bai J. Lian W. (2021). An attention-based CNN-BiLSTM hybrid neural network enhanced with features of discrete wavelet transformation for fetal acidosis classification. Expert Syst. Appl. 186, 115714. 10.1016/j.eswa.2021.115714

[B23] Ma N. Zhang X. Zheng H. T. Sun J. (2018). “Shufflenet V2: Practical guidelines for efficient cnn architecture design,” in Lecture notes in computer science (including subseries lecture notes in artificial intelligence and lecture notes in bioinformatics) 11218 LNCS (Munich, Germany: Springer, Cham), 122–138. 10.1007/978-3-030-01264-9_8

[B24] Marques J. A. L. Cortez P. C. Do Vale Madeiro J. P. Fong S. J. Schlindwein F. S. De Albuquerque V. H. C. (2019). Automatic cardiotocography diagnostic system based on Hilbert transform and adaptive threshold technique. IEEE ACCESS 7, 73085–73094. 10.1109/ACCESS.2018.2877933

[B25] Palomaki O. Luukkaala T. Luoto R. Tuimala R. (2006). Intrapartum cardiotocography - the dilemma of interpretational variation. J. Perinat. Med. 34 (4), 298–302. 10.1515/jpm.2006.057 16856819

[B26] Sandler M. Howard A. Zhu M. Zhmoginov A. Chen L. C. (2018). “MobileNetV2: Inverted residuals and linear bottlenecks,” in Proceedings of the IEEE computer society conference on computer vision and pattern recognition (Salt Lake City, UT, United States: IEEE), 4510–4520. 10.1109/cvpr.2018.00474

[B27] Spairani E. Daniele B. Signorini M. G. Magenes G. (2022). A deep learning mixed-data type approach for the classification of FHR signals. Front. Bioeng. Biotechnol. 10, 887549. 10.3389/fbioe.2022.887549 36003538 PMC9393210

[B28] Spilka J. Georgoulas G. Karvelis P. Chudaek V. Stylios C. D. Lhotska L. (2014). “Discriminating normal from "abnormal" pregnancy cases using an automated FHR evaluation method,” in Lecture notes in computer science (including subseries lecture notes in artificial intelligence and lecture notes in bioinformatics) (Ioannina, Greece: Springer, Cham), 521–531. 10.1007/978-3-319-07064-3_45

[B29] Vayssiere C. Haberstich R. Sebahoun V. David E. Roth E. Langer B. (2007). Fetal electrocardiogram ST-segment analysis and prediction of neonatal acidosis. Int. J. Gynecol. Obstetrics 97 (2), 110–114. 10.1016/j.ijgo.2007.01.003 17368461

[B30] Yilmaz E. (2016). Fetal state assessment from cardiotocogram data using artificial neural networks. J. Med. Biol. Eng. 36 (6), 820–832. 10.1007/s40846-016-0191-3

[B31] Zarmehri M. N. Castro L. Santos J. Bernardes J. Costa A. Santos C. C. (2019). On the prediction of foetal acidaemia: A spectral analysis-based approach. Comput. Biol. Med. 109, 235–241. 10.1016/j.compbiomed.2019.04.041 31085380

[B32] Zeng R. Lu Y. Long S. Wang C. Bai J. (2021). Cardiotocography signal abnormality classification using time-frequency features and ensemble cost-sensitive SVM classifier (vol 130, 104218, 2021). Comput. Biol. Med. 134, 104466. 10.1016/j.compbiomed.2021.104466 33484945

[B33] Zhang X. Zhou X. Lin M. Sun J. (2018). “ShuffleNet: An extremely efficient convolutional neural network for mobile devices,” in Proceedings of the IEEE computer society conference on computer vision and pattern recognition (Salt Lake City, UT, United States: IEEE), 6848–6856. 10.1109/cvpr.2018.00716

[B34] Zhao Z. Zhang Y. Comert Z. Deng Y. (2019). Computer-aided diagnosis system of fetal hypoxia incorporating recurrence plot with convolutional neural network. Front. Physiology 10, 255. 10.3389/fphys.2019.00255 PMC6422985 30914973

[B35] Zhao Z. Zhang Y. Deng Y. (2018). A comprehensive feature analysis of the fetal heart rate signal for the intelligent assessment of fetal state. J. Clin. Med. 7 (8), 223. 10.3390/jcm7080223 30127256 PMC6111566

[B36] Zheng B. Liu Y. He K. Wu M. Jin L. Jiang Q. (2021). Research on an intelligent lightweight-assisted pterygium diagnosis model based on anterior segment images. Dis. Markers 2021, 7651462. 10.1155/2021/7651462 34367378 PMC8342163

