# Supplementary material for: A lightweight fetal distress-assisted diagnosis model based on a cross-channel interactive attention mechanism
Source: Front Physiol. 2023 Mar 6;14:1090937. doi: 10.3389/fphys.2023.1090937 (PMC10025355; doi:10.3389/fphys.2023.1090937)
Supplement: Supplementary file 1 [file DataSheet1.PDF]

Supplementary Material

1 Supplementary Figures and Tables

1.1 Supplementary Figures

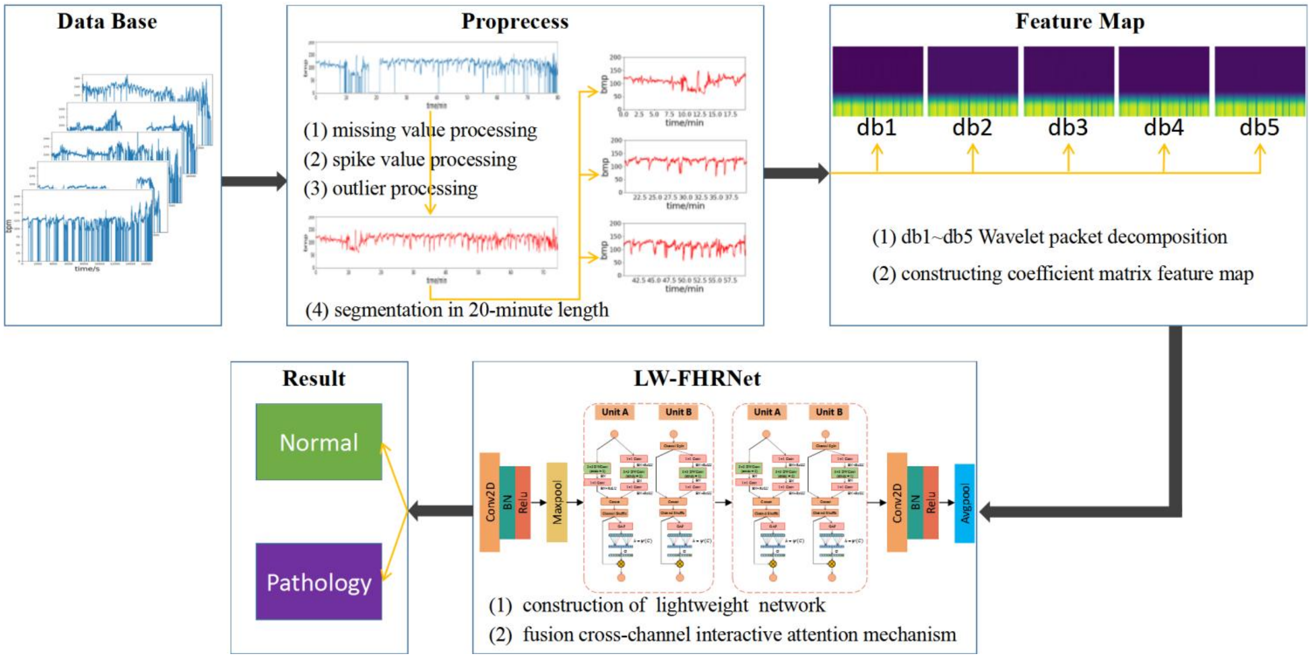

Supplementary Figure 1. Description of the architecture for the proposed lightweight network-based fetal distress assisted-diagnosis model.

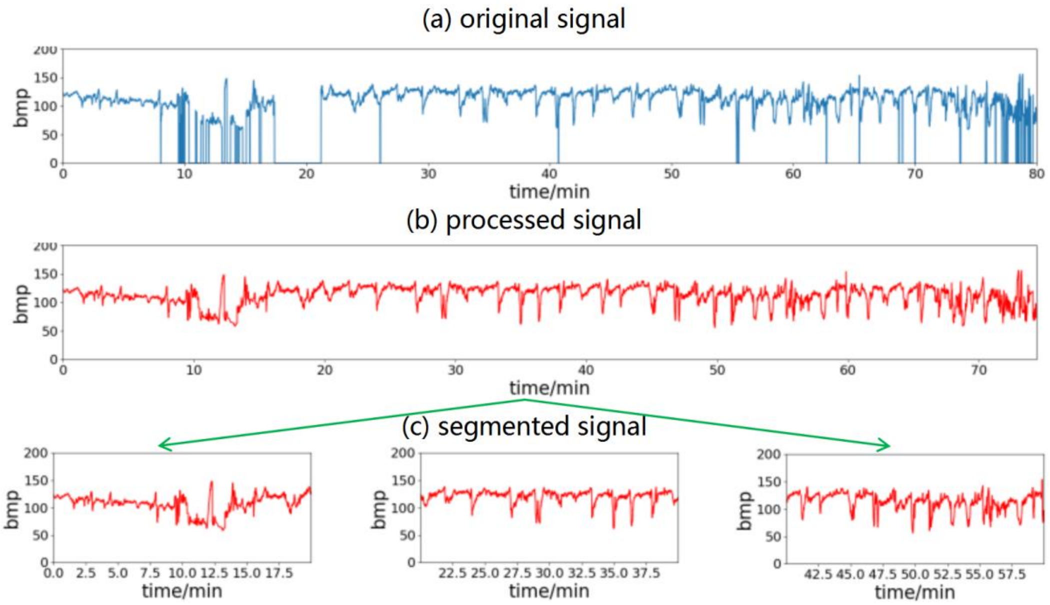

Supplementary Figure 2. FHR signal preprocessing process. Remove spikes and missing values of the original signal, then divide into segments of 20-minute length. (a) the original signal, (b) processed signal, (c) segmented signal.

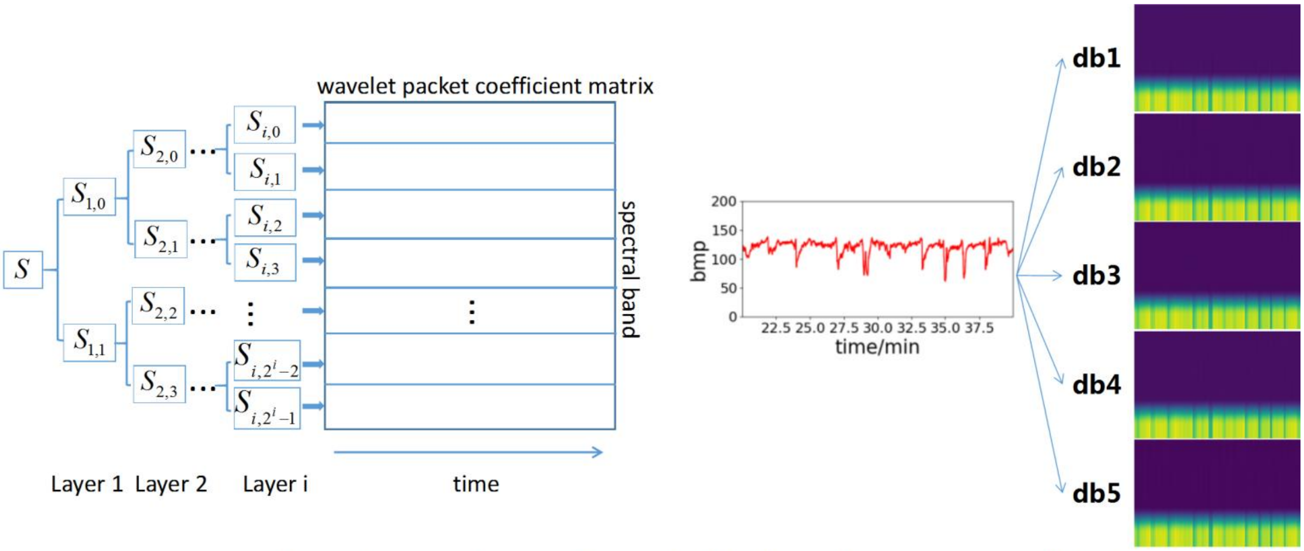

(a) Construction of wavelet packet coefficient matrix (b) A FHR signal segment and its db1~db5 feature maps

Supplementary Figure 3. Construction of feature maps based on wavelet packet coefficient matrix. (a) Construction of wavelet packet coefficient matrix; (b) Construction of db1~db5 feature map.

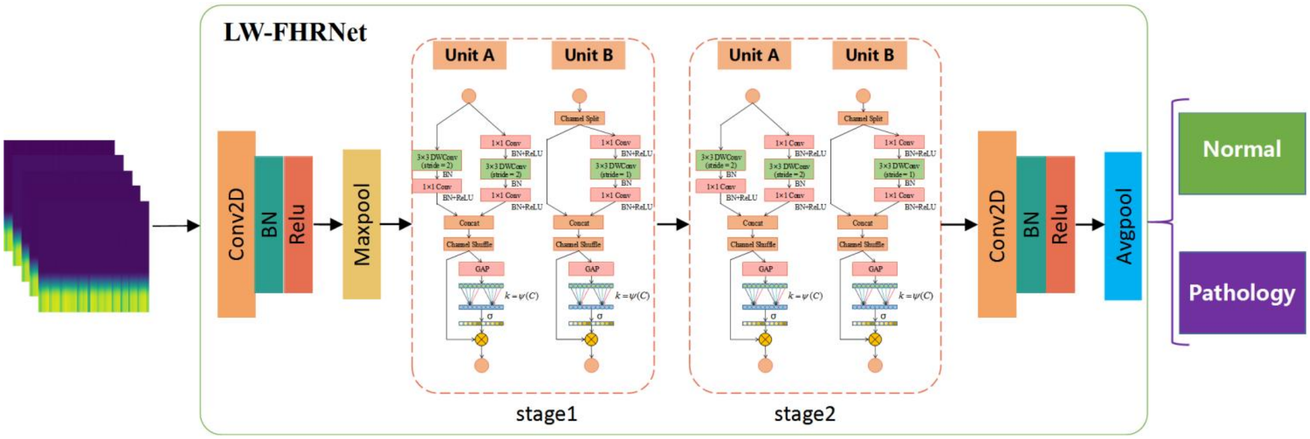

Supplementary Figure 4. The structure of LW-FHRNet. Notes: Conv2D: Convolution2D; BN: Batch Normalization; Maxpool: Max pooling; Avgpool: Average pooling.

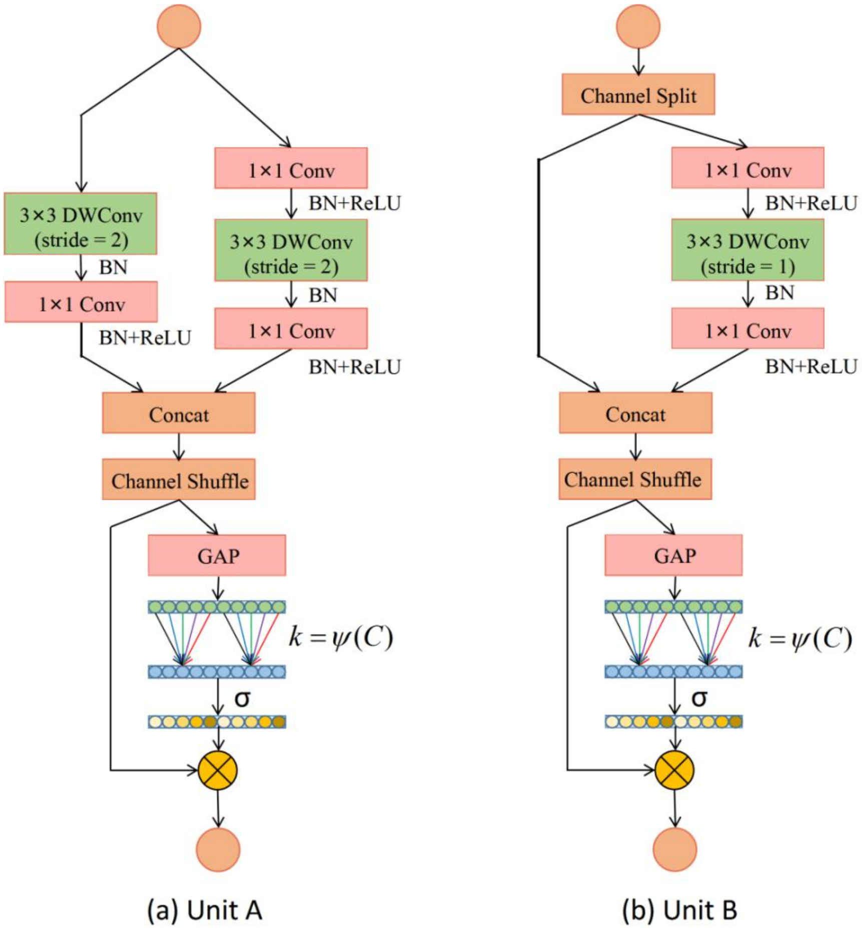

Supplementary Figure 5. Detailed description of the ECA-Shuffle unit. Notes: DWConv: Depthwise separable convolution; Conv: convolution; BN: Batch Normalization; GAP: Global Average Pooling

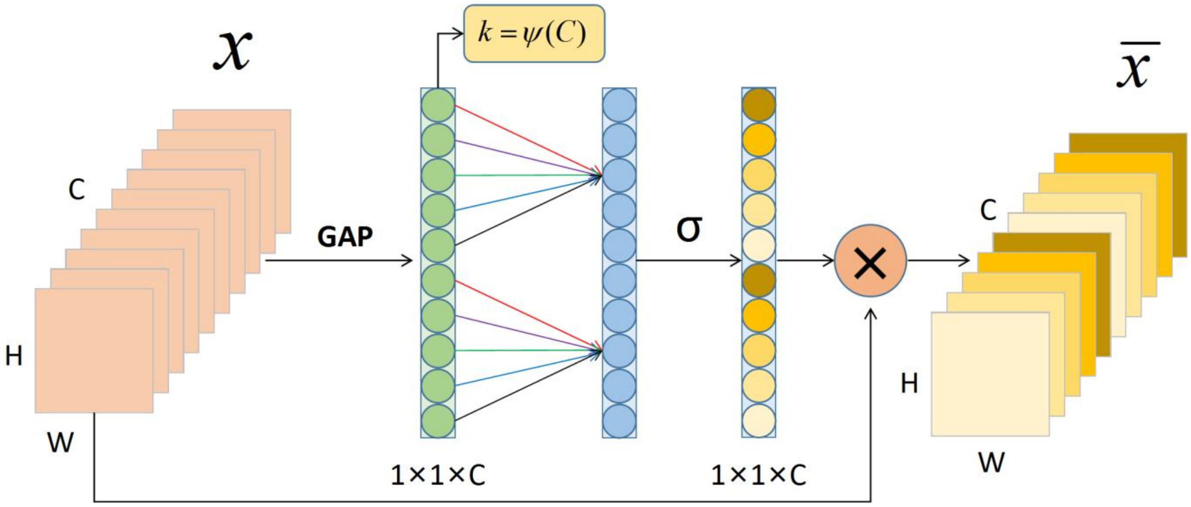

Supplementary Figure 6. The cross-channel interactive attention module. Notes: GAP: Global Average Pooling; C: Channel dimension; H: Height; W: Width

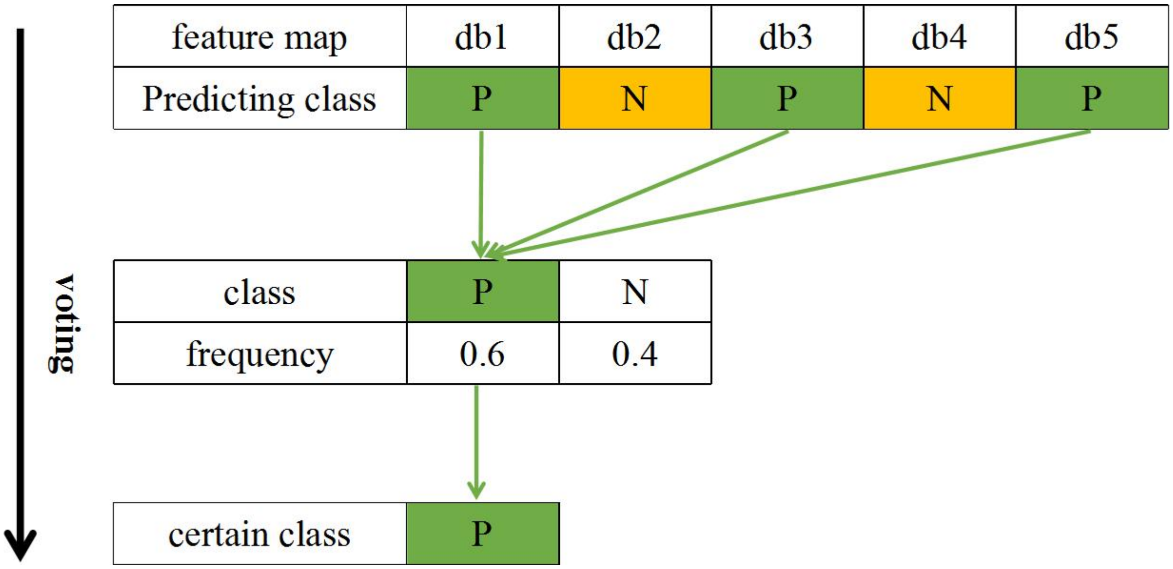

Supplementary Figure 7. An example of the category voting process. Notes: P: Positive; N: Negative.

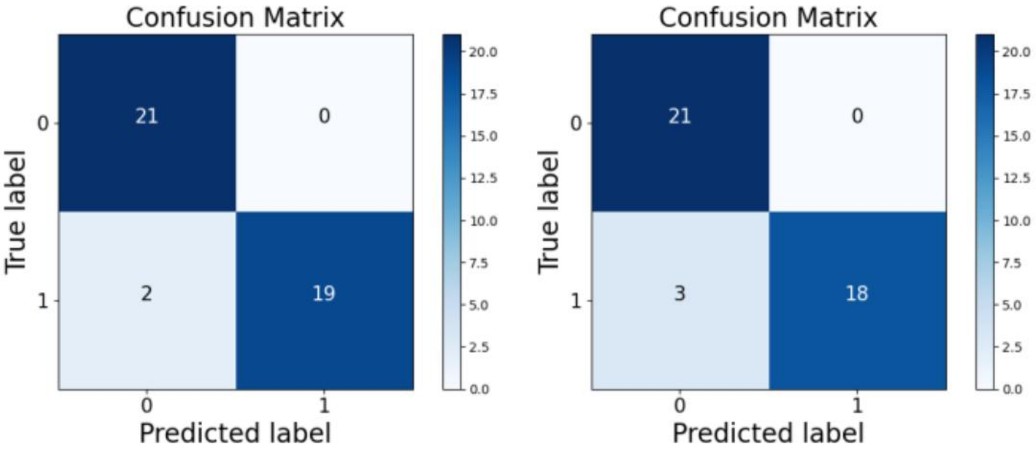

(a) LW-FHRNet (b) LW-FHRNet-without-ECA

Supplementary Figure 8. Confusion matrix. (a) the proposed LW-FHRNet, (b) the proposed LW-FHRNet without the ECA module.

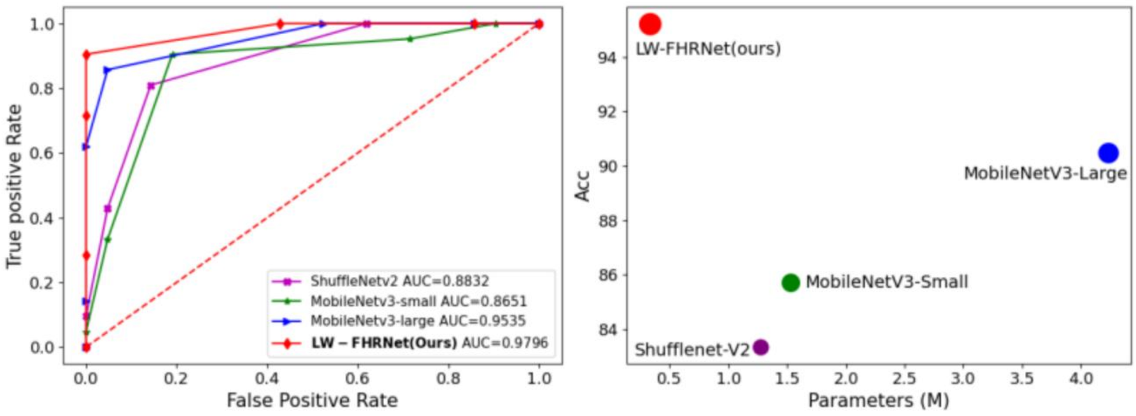

(a) (b)

Supplementary Figure 9. Classification performance of different lightweight models. (a) ROC curves of different lightweight models; (b) Acc and parameters of different lightweight models, where green, blue, purple, and red refer to MobileNetV3-Small, MobileNetV3-Lagre, ShuffleNetV2, and LW-FHRNet (Ours).

1.2 Supplementary Tables

Supplementary Table 1 | The structure parameter information of LW-FHRNet.

Layer	Output size	Kernel size	Output channel
Input	224 x 224	-	3
Conv	112 x 112	3 x 3	24
MaxPool	56 x 56	3 x 3	
Stage1	28 x 28	-	116
Stage2	14 x 14	-	232
Conv	14 x 14	1 x 1	1024
AvgPool	1 x 1	14 x 14	
FC	-	-	1

Notes: The normalization and ReLU layers that follow each convolutional layer are not shown above because they do not change the output feature shape. Conv: convolutional layer; MaxPool: max pooling layer; AvgPool: average pooling layer; FC: fully connect layer; stage: ECA-Shuffle uint A + ECA-Shuffle uint B

Supplementary Table 2 | Details of LW-FHRNet classification algorithm.

Input: S^{train} training sample sets; L^{train} training label sets, S^{test} testing sample sets; L^{test} testing label sets;
Output: Prediction label $\widetilde{L}^{test}$ of the S^{test};

for dbi in $[db1, db2, db3, db4, db5]$ **do**
$F_{dbi}^{train} = PWT_{dbi}(S^{train})$ # $PWT_{dbi}(\cdot)$ is the wavelet packet decomposition based on the dbi wavelet basis functions
$L_{dbi}^{train} = L^{train}$
$F_{dbi}^{test} = PWT_{dbi}(S^{test})$
$L_{dbi}^{test} = L^{test}$
end for
# training procedure
Initialize parameters and weights
for i in [1, 2, 3, 4, 5] **do**
metrics = $LW-FHRNet(F_{dbi}^{train}, L_{dbi}^{train})$
Train the LW-FHRNet model by optimizing the loss function
end for
return model LW-FHRNet-best
# testing procedure
for i in [1, 2, 3, 4, 5] **do**
$L_{dbi}^{test} \xleftarrow{predict}$ LW-FHRNet-best(F_{dbi}^{test})
end for
$\widetilde{L}^{test} = vote(L_{db1}^{test}, L_{db2}^{test}, L_{db3}^{test}, L_{db4}^{test}, L_{db5}^{test})$ # $vote()$ is a voting function
return $\widetilde{L}^{test}$

Supplementary Table 3 | Performance comparison of feature maps constructed by different layers of wavelet packet decomposition.

Decomposition Level	Accuracy (%)	Precision (%)	Recall (%)	F1-Score (%)
layer 1	83.33	85.00	80.95	82.93
**layer 2**	**95.24**	**100**	**90.48**	**95.00**
layer 3	90.48	94.74	85.71	90.00
layer 4	80.95	84.21	76.19	80.00
layer 5	76.19	76.19	76.19	76.19

Supplementary Table 4 | Lightweight model performance comparison with and without the ECA module.

Model	Accuracy (%)	Precision (%)	Recall (%)	F1 Score (%)
**LW-FHRNet**	**95.24**	**100**	**90.48**	**95.00**
LW-FHRNet-without-eca	92.86	100	85.71	92.31

Supplementary Table 5 | Performance comparison of different lightweight models for fetal distress classification.

Network	Accuracy (%)	Precision (%)	Recall (%)	F1 Score (%)	Parameter (M)	Model Size (M)
MobileNetV3-Small	85.71	82.61	90.48	86.36	1.53	5.84
MobileNetV3-Large	90.48	94.74	85.71	90.00	4.23	16.13
ShuffleNet-V2	83.33	85.00	80.95	82.93	1.27	4.85
**LW-FHRNet(ours)**	**95.24**	**100**	**90.48**	**95.00**	**0.33**	**1.27**

Supplementary Table 6 | Comparison of recent studies on the prediction of fetal distress using the CTU-UHB database.

Author	Division criteria	Method	Performance(%)		
			Acc	Se	Sp

Comert et al. (2018)	PH ≤ 7.15	BFS, DWT + SVM	67.00	57.42	70.11
Fuentealba et al. (2019)	PH < 7.05; BDecf≥12<br>PH > 7.20; BDecf≥12	CEEMDAN, TV-AR + SVM	81.7	79.5	86.45
Zarmehri et al. (2019)	PH ≤ 7.05	FFT	/	63.60	80.10
Alsaggaf et al. (2020)	PH < 7.15	Morphological, linear, nonlinear, CSP + SVM	94.75	74.29	99.55
Zeng et al. (2021)	PH ≤ 7.05; BE ≤ -10	CWT, WTC, XWT + ECSVM	67.2	85.2	66.1
Liu et al. (2021)	PH ≤ 7.15	CNN-BiLSTM + Attention, DWT	71.71	75.23	70.82
Neeraj et al. (2021)	PH ≤ 7.15	1D CNN	99.09	/	/
**Ours**	**PH ≤ 7.05**	**WPT + LW-FHRNet**	**95.24**	**90.48**	**100**

Notes: BFS: basic feature set; DWT: discrete wavelet transform; CEEMDAN: complete ensemble empirical mode decomposition with adaptive noise; TV-AR: time-varying autoregressive; CSP: common spatial pattern; CWT: continuous wavelet transform; WTC: wavelet coherence; XWT: Cross-wavelet Transform; ECSVM: ensemble cost sensitive SVM; WPT: wavelet packet transform; Acc: Accuracy; Se: Sensitivity; Sp: Specificity .
